# Improved Wire Quality of Twinning-Induced Plasticity Steel During Wire Drawing Through Temperature Gradient with Warm Die

**DOI:** 10.3390/ma18061209

**Published:** 2025-03-08

**Authors:** Joong-Ki Hwang

**Affiliations:** School of Mechatronics Engineering, Korea University of Technology & Education, Cheonan 31253, Republic of Korea; jkhwang@koreatech.ac.kr; Tel.: +82-41-560-1642

**Keywords:** wire drawing, warm die, temperature gradient, drawability, twinning-induced plasticity steel

## Abstract

The drawability and microstructural homogeneity of twinning-induced plasticity (TWIP) steel were improved during the wire drawing process by utilizing a temperature gradient along the wire’s radial direction. The surface temperature of the wire increased by applying heat to the die during the drawing process, thereby creating a temperature gradient across the wire during wire drawing. The drawability of the wire subjected to the temperature gradient with warm die (WD) increased by approximately 33% compared to that of conventional wire drawing with cold die (CD). The higher temperature of about 300 °C at the surface region of the wire with the WD suppressed the twinning rate at the surface region owing to the increase in the stacking fault energy (SFE) from 34 to 55 mJ/m^2^, leading to a uniform twinning rate along the wire’s radial direction compared with the CD wire, finally resulting in the improvement of the homogeneity in the microstructure and mechanical properties of TWIP steel. As a result, the drawability of the TWIP steel improved. Therefore, the general conclusion was derived that controlling the SFE within the area of the workpiece by tailoring the temperature can improve the formability in TWIP steels during the plastic forming process.

## 1. Introduction

Significant efforts have been made in the transportation industry to enhance energy efficiency and driving performance through the development of advanced high-strength materials. For example, the steel industry is continuously striving to develop lightweight steel materials for cars. In particular, with the recent rise of electric vehicles, efforts to develop high-strength and lightweight steels have been intensified to prevent market loss to alternative materials, such as aluminum, carbon fiber-reinforced plastic, and magnesium. Among the various steels, twinning-induced plasticity (TWIP) steel has received great attention as an advanced automotive material owing to its exceptional combination of strength, ductility, and toughness stemming from the extensive deformation twinning [1,2,3] and/or dynamic strain aging (DSA) [4,5] generated during deformation.

Recently, Hwang et al. [6] also proposed that TWIP steels are good candidates for wire, rod, and bar products, such as fasteners, tie rod, bearings, and wire ropes, because TWIP steels meet their particular property requirements. However, their recent studies revealed that the formability during wire drawing, which is called drawability, in TWIP steels was not acceptable although TWIP steels have an outstanding tensile elongation [7]. Wire drawing is a widely utilized cold working process in the production of wires, rods, and bars across various industries [8]. In this process, the specimen is drawn through a series of specially designed dies, leading to a reduction in its cross-sectional area, while achieving the required shape and mechanical properties [9]. Drawability is generally defined as the ability of a material to undergo the wire drawing process and achieve a desirable shape in the absence of defect or fracture in the drawn wire. The authors [7] show that the surface area experiences greater strain compared to the center area during the wire drawing process, leading to strain inhomogeneity across the radial direction of the wire. This ultimately results in crack initiation at the surface due to ductility exhaustion, particularly caused by the depletion of twins. Hence, the twinning rate along the wire’s radial direction should be uniformly controlled to improve the drawability of TWIP steels. Meanwhile, it is well known that the twinning rate is mainly controlled by the stacking fault energy (SFE), and SFE is primarily controlled by temperature and chemical compositions [10,11,12,13,14]. Allain et al. [10] suggested that deformation twinning plays a key role in plasticity and strain hardening when the SFE ranges between 12 and 35 mJ/m^2^. Additionally, Saeed-Akbari et al. [12] reported that a SFE of 20 mJ/m^2^ represents the upper threshold for the formation of ε-martensite, while Curtze and Kuokkala [14] identified a SFE of 45 mJ/m^2^ as the lower threshold for the activation of slip. SFE increases as the material temperature rises, which in turn suppresses deformation twinning [15].

Meanwhile, during the plastic forming process, the material temperature increases due to the heat generated by deformation. The temperature increase during deformation is generally calculated based on the following simple equation [14,16]:

(1) $$\Delta T=\frac{\Delta Q}{\rho C_{p}}=\frac{\beta }{\rho c_{p}}\int_{\varepsilon_{1}}^{\varepsilon_{2}}\sigma d\varepsilon$$

where Δ*T*, *Q*, *ρ*, *c*_*p*_, and *β* refer to the temperature increase, heat energy, density, specific heat capacity, and fraction factor between mechanical work and heat energy, respectively. *β* is assumed to be 0.9 because little mechanical energy is stored in the deformed material as elastic energy [14,16]. Therefore, even when wire drawing is conducted at room temperature, the temperature of the wire increases due to the heat generated by plastic deformation. During the drawing process, the thermal gradient exists along the radial direction owing to the heating effect caused by the friction in the wire and die interface, which increases the surface temperature of the wire [17,18]. In other words, during drawing, the temperature at the surface region of the wire becomes higher compared to the center region. And then, with time the wire temperature becomes uniform along the radial direction since the high temperature in the surface region of the wire, owing to the friction, is transferred into the center region by conduction heat transfer and released to the atmosphere by convection and radiation heat transfers [19].

During wire drawing, the twinning rate increases along the wire’s radial direction since strain increases along the radial direction [20]. In order to make a similar twinning rate with the area of the wire, the twinning rate of the surface area needs to be decreased by increasing the SFE while tailoring the surface temperature. That is, the temperature gradient across the wire’s radial direction can promote a uniform twinning rate throughout the material, even in the presence of a strain gradient along the radial direction, leading to an increase in drawability in TWIP steels. However, no research has been reported on the influence of the temperature gradient on the wire drawing behavior in TWIP steels.

Therefore, this study investigates the influence of the temperature gradient within the wire on the mechanical properties and microstructure of TWIP steel during the wire drawing process, aiming to enhance its drawability. In this study, the die temperature was adjusted to establish a temperature distribution across the wire. Specifically, by applying heat to the die during wire drawing, the temperature at the surface region of the wire increased, thereby creating a temperature gradient across the wire during wire drawing. Finite element analysis (FEA) and hardness testing were performed to investigate the temperature and strain distributions along the radial direction of the deformed drawn wire. Furthermore, the mechanical properties of the drawn wire in relation to its area were validated using microstructural evolution with electron backscatter diffraction (EBSD) techniques.

## 2. Experimental and Numerical Simulation Procedures

### 2.1. Material Preparation of TWIP Steel

A 50 kg high-manganese steel was prepared using a vacuum induction furnace. Table 1 gives the chemical compositions of the present TWIP steel analyzed by a spark optical emission spectrometer. It also presents the calculated SFE of the steel based on the thermodynamic models proposed by Saeed-Akbari et al. [12] and Dumay et al. [13]. The calculated SFE is 29.5 mJ/m^2^. This value is expected to promote both deformation twinning and slip during plastic deformation at room temperature. Conversely, martensitic transformation is anticipated to be inhibited at room temperature [10,21]. It has been established that the γ phase is exclusively present when SFE is 15 mJ/m^2^ or higher [14,22].

For hot rolling, the cast ingot was machined into a billet with a cross section of 125 mm × 125 mm using a water jet machine. The billet was kept at 1200 °C for 12 h in a furnace for the homogenization and then rolled at temperature above 950 °C using several rolling mills. The final thickness of the hot-rolled plate is 20 mm. The hot-rolled plate was cooled naturally by ambient air on the cooling bed.

### 2.2. Wire Drawing Test Under CD and WD Process Conditions

The hot-rolled plate was processed into cylindrical bars with a diameter of 13 mm and a length of 400 mm for use in the wire drawing test. The drawing tests were carried out at a constant pulling speed of 50 mm/s, a semi-die angle of 6°, and average reduction in area (RA) per pass of 20% at room temperature (RT, 26 °C) using a single-pass draw bench machine. The detailed process conditions are listed in Table 2. The RA per pass and nominal drawing strain (*ε*_n_) of the drawn wire are obtained as follows:

(2) $$RA=\frac{A_{i}-A_{f}}{A_{i}}\times 100\;\left(\%\right)$$

(3) $$\varepsilon_{n}=2ln(\frac{D_{i}}{D_{f}})$$

where *A* and *D* represent the wire’s area and diameter, respectively. The subscripts i and f indicate the initial and final values of the wire, respectively. The bars were drawn two ways: wire drawing with cold die (CD) and wire drawing with warm die (WD). The CD process refers to a conventional wire drawing process at RT. In the case of the WD experiment, a torch was used to heat the drawing die to maintain a temperature of approximately 400 °C during the experiment. Five repeated tests were conducted for each condition. It is worth noting that it is not easy to keep the die temperature at 400 °C manually. The pyrometer measurements of the die showed a temperature variation of ±35 °C during the test.

### 2.3. Measurements of Mechanical Properties and Microstructure

**Figure 1:**
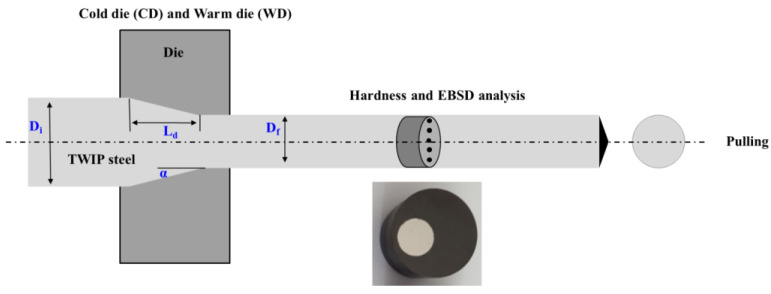
Schematic diagram of hardness and microstructure analysis for drawn wire.

Tensile specimens of cylindrical shapes were machined using a lathe aligned with the rolling direction. The gauge diameter and length are 5 mm and 25 mm, respectively. The gauge length-to-diameter ratio was set to 5.0. The specimens were strained at an initial strain rate of 10^−3^ s^−1^ using an Instron at RT. The strain was obtained using a mechanical extensometer. Vickers hardness (HV) tests were conducted on the cross-section of the drawn wire, perpendicular to the drawing direction, using a 1 kg load and holding time of 15 s, as shown in Figure 1. Approximately 29 measurements were performed at each condition.

Microstructural analysis was performed using EBSD. The specimens were initially ground sequentially with commercial silicon carbide papers up to 2000 grit using an automated polishing system. Following this, they were polished with diamond pastes of varying sizes, ranging from 6 μm to 1 μm. For the final surface preparation, a colloidal silica suspension was applied for approximately 1.2 ks to achieve EBSD-ready surfaces. The EBSD data acquisition was carried out using a field-emission scanning electron microscope (SEM) equipped with a TexSEM Laboratories EBSD system (AMETEK, Berwyn, PA, USA), operating at an acceleration voltage of 20 kV. The hot-rolled samples were analyzed over an area of 140 μm × 140 μm with a step size of 0.15 μm, whereas the drawn wire samples were examined over a 50 μm × 120 μm region with a step size of 0.1 μm to assess twin density. A tilt angle of 70° was applied during the analysis, and both the center and surface regions of the specimen were investigated. Microstructures were also measured on the cross-section of the drawn wire, perpendicular to the drawing direction, as shown in Figure 1. The acquired EBSD data were further processed using orientation imaging microscopy (OIM, version 8) software.

### 2.4. Finite Element Analysis of Wire Drawing Process

During the wire drawing process, variations in stress, strain, and temperature arise within the wire, leading to an inhomogeneous distribution [23]. To gain a comprehensive understanding of the complex strain and temperature distributions under CD and WD process conditions, FEA was utilized, providing valuable insights into metal forming processes. The wire drawing simulation was conducted using the commercial software DEFORM (Version 13.1). An axisymmetric module was employed due to the symmetry characteristics of the drawing process.

For the FEA, the deformation behavior of the wire is necessary during wire drawing. In this study, the flow curve of the wire was derived from the tensile stress–strain data of hot-rolled TWIP steel. The material was considered isotropic, and its deformation behavior was described using the strain hardening coefficient (*K*) and exponent (*n*). The relationship between these parameters can be represented by Hollomon’s law, which is expressed as follows:

(4) $$\sigma =K\epsilon^{n}$$

The initial wire temperature was assumed to be 26 °C. Meanwhile, die was modeled as a rigid body, meaning that the die was assumed to be an undeformable component. The die temperature was assumed to be 26 °C under the CD process condition. While in the WD process condition, the die temperature was set to 400 °C. A shear friction factor of 0.1765 was adopted based on previous research [24]. The initial diameter of the wire, drawing speed (*V*), RA per pass, and semi-die angle (*α*) were set to 13 mm, 50 mm/s, 20%, and 6°, respectively, which corresponds to the experimental conditions listed in Table 2. During the drawing process, stain rate ($\dot{\varepsilon }$) is calculated from the product of *V* and ε_N_ divided by the deformation zone length (L_*d*_), as follows [10].

(5) $$\dot{\varepsilon }=\frac{\varepsilon_{N}\cdot V}{L_{d}}$$

L_*d*_ is obtained based on the trapezoidal-shaped deformation zone of conical die (as shown in Figure 1) as follows:

(6) $$L_{d}=\frac{D_{i}-D_{f}}{2tan\alpha }$$

The calculated $\dot{\varepsilon }$ of the wire was approximately 1.69 s^−1^. The thermal conductivity (*k*) of the wire and die were assigned based on references [25,26]. For example, the *k* of TWIP steel was determined using the following equation [25].

*k* = 13.7 + 0.013 *T* (°C) (7)

In addition, the *k* of die with a tungsten carbide was considered to be a constant value of 70 W/m°C [26].

To enhance computational efficiency, only half of the wire and die was modeled, taking advantage of the symmetry inherent in the conventional wire drawing process [27]. Approximately 10,000 brick-type elements for the workpiece and around 2000 elements for the die were used based on the previous result [28].

## 3. Results

### 3.1. Microstructure Evolution

**Figure 2:**
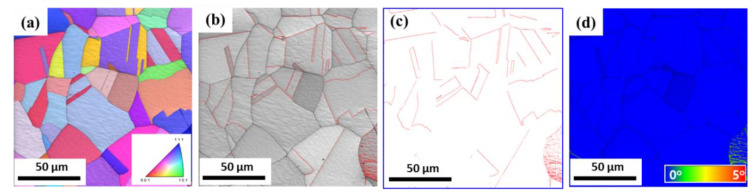
(**a**) EBSD IPF, (**b**) IQ with Σ3 twin boundaries, (**c**) twin boundaries, and (**d**) KAM maps of hot-rolled present TWIP steel.

Figure 2 presents the microstructure of the hot-rolled (*ε*_N_ = 0.0) specimen using EBSD. The inverse pole figure (IPF) map, image quality (IQ) map with Σ3 twin boundaries, twin boundaries map, and Kernel average misorientation (KAM) map were analyzed. The Σ3 twin boundaries were determined by the misorientation angle of 57° < θ < 63° [29,30] and presented as a red line in Figure 2b,c. It is known that the spatial gradient in orientation can reflect the geometrically necessary dislocation (GND) density and/or plastic deformation [31]. In other words, the KAM value can be considered as an indicator of GND density. Annealing twins were observed; however, deformation twins were not detected in the hot-rolled TWIP steel. The microstructure was composed of equiaxed grains with an average grain size of 27.5 μm. In addition, no plastic deformation was observed based on the KAM map (Figure 2d).

**Figure 3:**
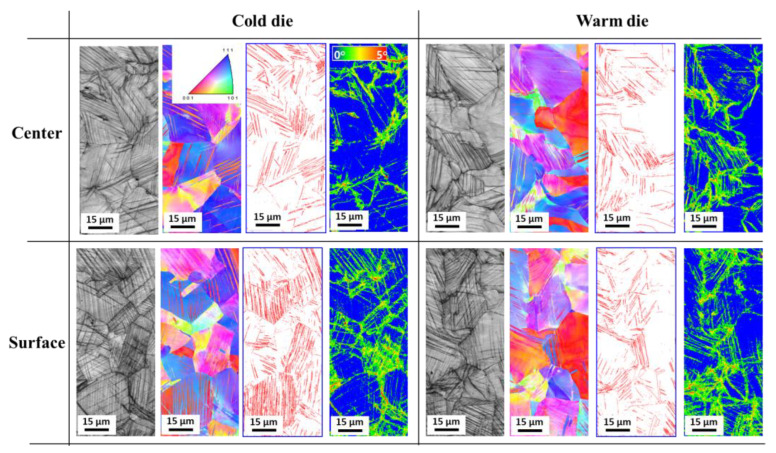
Comparison of IQ, IPF, twin boundaries, and KAM maps at nominal drawing strain of 0.22 under CD and WD processes.

**Figure 4:**
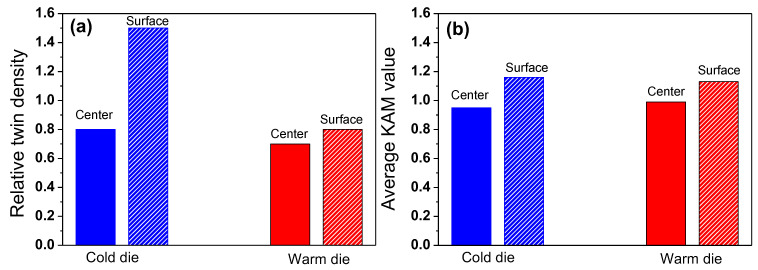
Comparison of (**a**) relative twin density and (**b**) average KAM values at nominal drawing strain of 0.22 under CD and WD processes.

Figure 3 compares the IQ, IPF, twin boundaries, and KAM maps of the deformed wires at a drawing strain of 0.22 subjected to the CD and WD processes. Compared with the specimen under the hot-rolled state, it can be observed that deformation twins and GND appear at the drawn wires subjected to both CD and WD processes. The twin density in the center region of the wire deformed by the CD process was comparable to that observed in the WD process, whereas the CD wire exhibited a larger twin density compared with the WD wire at the surface region. To compare the twin density quantitatively, the relative twin density (R_*twin*_) was defined as follows:

(8) $$R_{twin}=\frac{b_{twin}}{R_{mea}}(mm/{mm}^{2})$$

where *b*_*twin*_ refers to the total length of the twin boundary in a measured area and *R*_*mea*_ is the total measured area. The result of the R_*twin*_ is shown in Figure 4a. The difference of the R_*twin*_ between the center and surface area was clearly observed in the CD wire. This indicates that deformation twinning was initiated earlier in the surface region than in the center region. Conversely, the R_*twin*_ of the WD wire was more homogeneous at the center and surface regions than those of the CD wire.

Meanwhile, the KAM maps showed similar results in the areas between the CD and WD wires: the surface region exhibited a higher KAM value than the center region regardless of the process conditions, which is well summarized in Figure 4b.

### 3.2. Mechanical Properties

**Figure 5:**
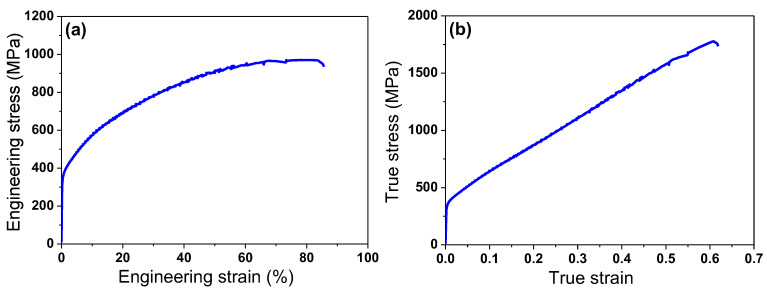
(**a**) Engineering and (**b**) true stress–strain curves of hot-rolled TWIP steel.

Figure 5 presents the tensile stress–strain curves of the hot-rolled TWIP steel. This material demonstrated an excellent balance of strength, ductility, and toughness: a tensile strength exceeding 900 MPa and a total elongation surpassing 80%. Additionally, it exhibited a relatively low yield strength compared with a high tensile strength and small post-necking elongation compared with large uniform elongation, which are commonly observed features of high-manganese TWIP steels [2,15]. Serrated flow behavior, attributed to DSA, was observed at high strain levels, a phenomenon commonly reported in C-containing TWIP steels [4,32,33].

**Figure 6:**
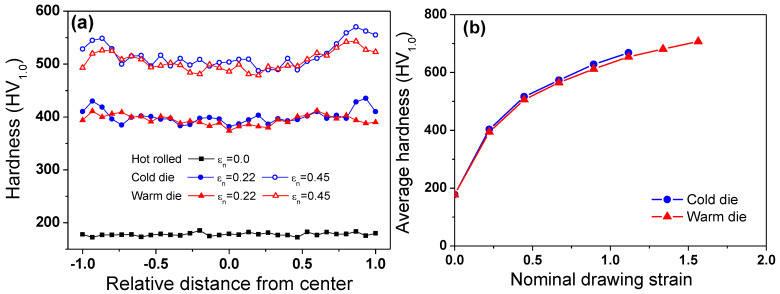
Comparison of variations in measured (**a**) hardness profiles along wire’s radial direction at nominal drawing strain of 0.22 and 0.45, and (**b**) average hardness with nominal drawing strain.

The hardness of the specimens subjected to the CD and WD processes was compared, as shown in Figure 6. The comparison of the hardness profile along the wire’s radial direction revealed that the hardness values of the center area were similar in the two processes, whereas the hardness of the surface area was different: the hardness of the WD wire was lower than that of the CD wire. Consequently, the WD wire had more uniform hardness distribution along the radial direction compared to the CD wire. This observation aligns with the findings on microstructural evolution (Figure 3). The CD wire exhibited a slightly higher average hardness than the WD wire due to the softening effect at the surface area of the WD wire, as shown in Figure 6b. Meanwhile, in the case of the high-manganese TWIP steel, the mechanical properties should be evaluated considering the martensitic transformation during plastic deformation. The calculated SFE of the present TWIP steel is 29.5 mJ/m^2^. Saeed-Akbari et al. [12] showed that a SFE of 20 mJ/m^2^ is the upper limit for ε-martensite transformation. Kim and De Cooman [22] revealed that 13 mJ/m^2^ is the upper limit of the SFE for *ε*-martensite transformation. Allain et al. [10] suggested that ε-martensite transformation occurrs when the SFE is lower than 18 mJ/m^2^. Zambrano [34] reported that deformation twinning primarily occurs at a SFE of 20–40 mJ/m^2^. Therefore, the TWIP steel in the present study was expected to promote the formation of deformation twin and dislocation glide rather than martensitic transformation. In addition, the variation in the chemical composition and structure along the radial direction of the drawn wire is an important factor that could influence the local SFE [35]. In this study, to exclude the influence of compositional or structural variations in the radial direction of the wire, heat treatment was performed at 1200 °C for 12 h, followed by about 84% hot rolling. Therefore, the effects of these factors were not considered in this study. However, it is presumed that modifying the composition or structure in specific regions of the wire could improve the drawability, which could be a potential area for further research.

**Figure 7:**
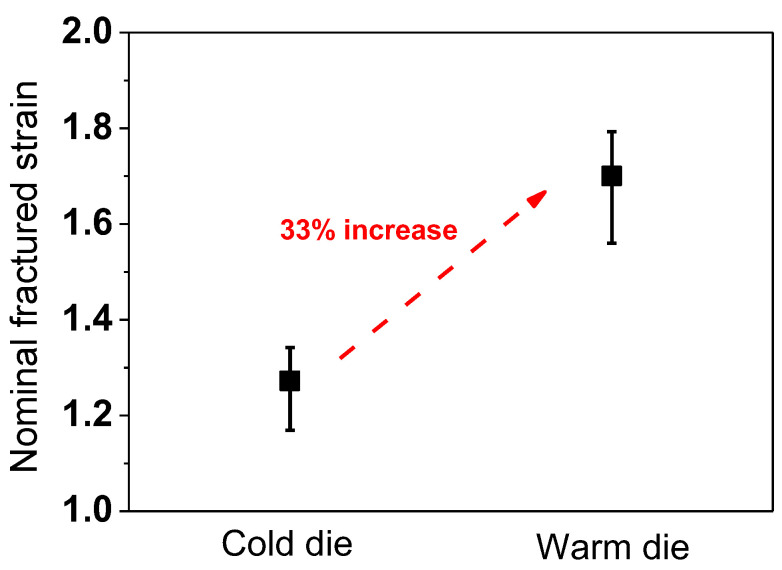
Comparison of measured fractured strain of wire under CD and WD process conditions.

To evaluate the drawability of the two drawing processes, fractured nominal drawing strains were compared, as shown in Figure 7. Interestingly, the wires subjected to the WD process had higher drawability than those of the CD process: the WD process increased the drawability by about 33% compared to the conventional CD process, which is greatly beneficial in industrial fields.

### 3.3. Numerical Analysis

**Figure 8:**
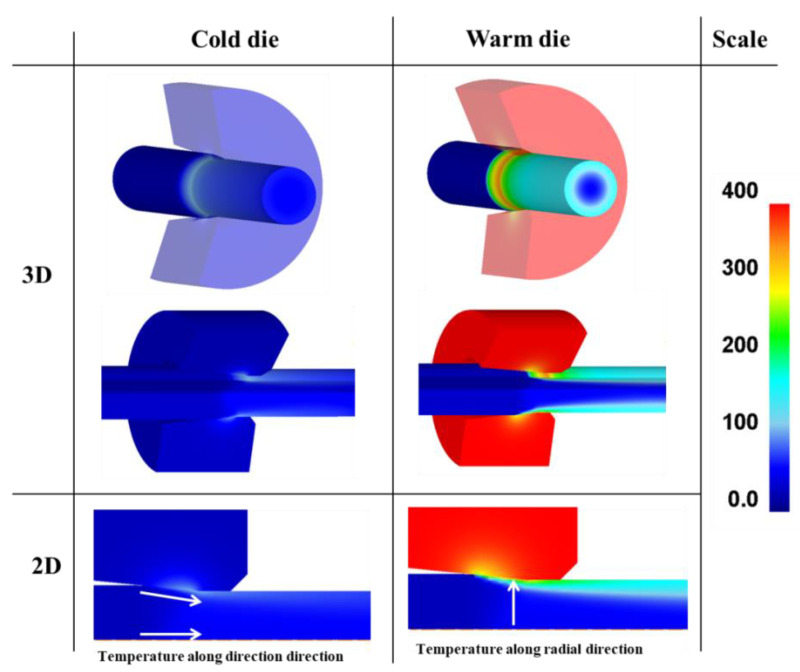
Comparison of temperature contours between CD and WD processes based on FEA.

Figure 8 illustrates the FEA results of the temperature distribution during the first drawing pass for both the CD and WD drawing processes. The values of *K* and *n* used in Equation (4) for the numerical simulation were obtained through the curve fitting of the true strain–stress curve in Figure 5b, yielding the equation as follows: *σ* = 2276*ε*^0.56^ MPa. To obtain more accurate results, it is necessary to determine and use the tensile curve based on the temperature and strain rate. However, within the temperature and strain rate range of this study, the analysis was conducted under the assumption that these factors would not significantly affect the flow stress of the material. The validation of the numerical results in this study was adapted from the results conducted by the author in previous research [36].

**Figure 9:**
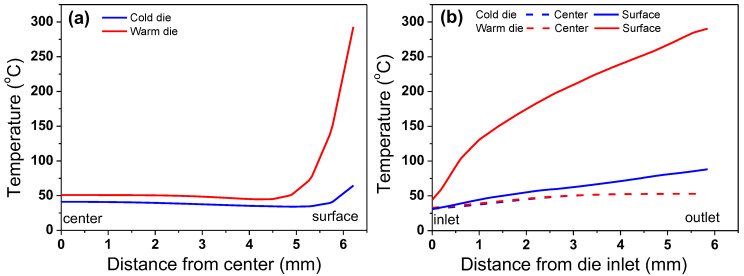
Comparison of temperature distribution (**a**) along radial direction which is depicted as white arrow in Figure 8 and (**b**) along drawing direction which is depicted as white arrow in Figure 8.

Figure 9 provides a comparison of the numerically simulated temperature profiles along the wire’s radial and drawing directions under the CD and WD process conditions, derived from the temperature contours in Figure 8. Under the CD process, a temperature gradient was observed across the wire’s radial direction, primarily due to the friction effect at the surface region between the wire and die, which led to a sharp increase in surface temperature [37]. Compared to the CD process conditions, it can be observed that the wire surface temperature was significantly higher under the WD process conditions due to the high die temperature. The increased die temperature results in a rise in the wire surface temperature due to the heat transfer from the die to the wire during the drawing process. On the other hand, the temperature at the center region of the wire remained almost the same regardless of the process conditions, as shown in Figure 9b. This indicates that the die temperature primarily affects the regions near the wire surface, leading to an increased temperature gradient along the wire’s radial direction, as shown in Figure 9a.

**Figure 10:**
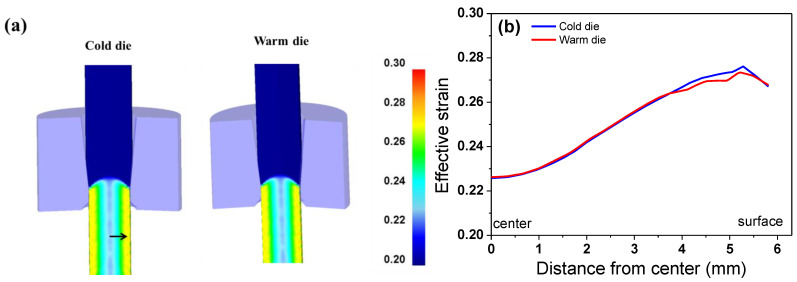
Comparison of (**a**) strain contours and (**b**) strain profiles along wire’s radial direction based on FEA.

Figure 10a compares the contour of the effective strain between the CD and WD wires. For a better comparison, the profiles of the strain distribution of the drawn wire were compared based on the line profile depicted as a black arrow in Figure 10a. Figure 10b presents the strain profiles along the wire’s radial direction. The wires exhibited the highest effective strain near the surface and the lowest at the center, aligning with previously findings [20,38]. It can be observed that the effective strain curves are similar regardless of the process conditions, indicating that the die temperature does not affect the effective strain distribution of the wire during drawing.

## 4. Discussion

The WD process improved the homogeneity of the mechanical properties and microstructure along the wire’s radial direction and enhanced the drawability of the TWIP steel during wire drawing. The reason why the WD wire exhibits more uniform microstructure and mechanical properties in the radial direction compared to the CD wire is closely related to the twinning behavior across the wire radius. In conventional wire drawing processes, the strain magnitude is higher at the surface region compared with the center region (Figure 10), and the surface region is subjected to a more complex stress state [7]. As a result, the surface region of the wire shows a higher R*_twin_* compared with the center region under conventional wire drawing process with CD, as shown in Figure 4a, leading to the difference in the mechanical properties across the wire radius (Figure 6a). Meanwhile, it should be noted that the microstructure and mechanical properties of metallic materials are influenced by the grain orientation [39,40,41,42]. For example, twinning behavior in TWIP steels depends on the grain orientation [41,42], which is generally explained by the Schmid factor. Deformation twins occurred in the grains close to <110> and <111> under the tensile stress [43]. In contrast, deformation twins occurred in the grains around <100> under the compressive stress [44]. In the case of the drawn wire at the center area, only tensile stress was applied during wire drawing; therefore, deformation twins primarily formed in the grains close to <110> and <111>. In contrast, deformation twins formed in all grains at the surface area of the drawn wire due to both the stress states of tension and compression, resulting in a higher R*_twin_* at the surface area of the drawn TWIP steel wire. This is one of the reasons why the CD wire exhibited a greater R*_twin_* at the surface area compared to the center area.

**Figure 11:**
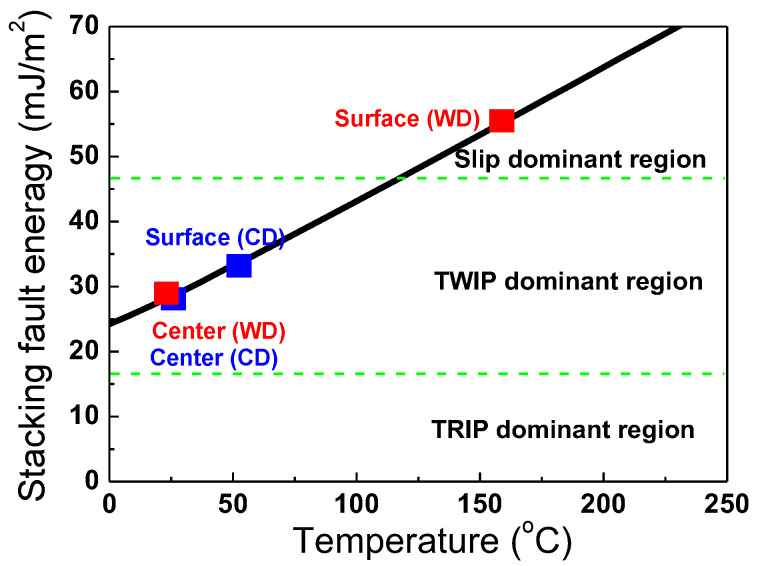
Calculated stacking fault energy of CD and WD wire with area as function of deformation temperature.

The high R*_twin_* at the surface region also leads to early twin exhaustion, which ultimately limits the ductility of the drawn wire [7]. By utilizing the WD process, the surface temperature of the wire increases, which in turn raises the SFE of the surface region, thereby suppressing twin formation, as shown in Figure 11. It is well known that an increase in SFE in TWIP steel suppresses twinning and promotes slip deformation [14]. Even when high stress is applied to the surface region during the drawing process, as shown in Figure 10, the suppression of the twinning by increasing the SFE at the surface region allows for a more uniform twin distribution across the wire radius (Figure 4a).

**Figure 12:**
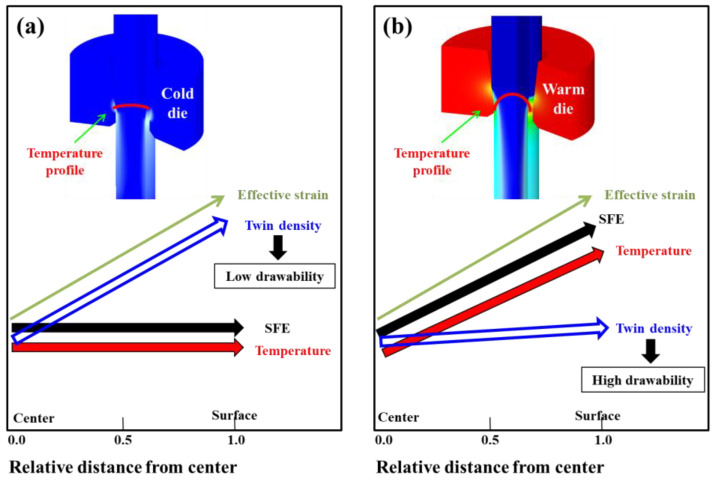
Schematic diagram of main variables along radial direction of TWIP steel wires during drawing process: (**a**) conventional wire drawing with CD and (**b**) proposed design concept for wire drawing with WD.

Figure 12 presents an overall schematic diagram of the improved drawability achieved through the WD process. Utilizing the WD process conditions enables the control of the temperature, SFE, twinning behaviors, and mechanical properties at the surface region of the wire. Ultimately, it can prevent crack formation caused by ductility exhaustion at the wire surface, thus enhancing the drawability of the wire. The influence of the twinning rate on the cup formability of TWIP steels was also previously investigated. Chin et al. [45] demonstrated that cracks tend to initiate on the cup side, where the density of deformation twins is higher than in other regions due to greater strain accumulation. Similarly, Renard and Jacques [46] found that fracture in tensile specimens occurs when the twin volume fraction reaches saturation. Meanwhile, the amount of plastic deformation at the center and surface regions of the CD and WD wires is similar based on GND density, as shown in Figure 4b. In other words, both the CD and WD wires experienced similar stress in regions despite the different twinning behaviors. This means that the different formabilities of the CD and WD wires are closely correlated with their twinning behavior. Considering both the present findings and previous studies [7], it can be inferred that the delayed saturation of deformation twins in the surface region of the wire contributes to an increased fracture strain in the WD wire, thereby enhancing its drawability. Additionally, according to the general theory on temperature and plastic deformation in metals, at elevated temperatures, the grain boundaries become slightly unstable, promoting grain deformability, which can result in better drawability. Based on the above results, the general conclusion was reached that controlling the SFE within an area by tailoring the temperature can improve the formability in TWIP steels during the plastic forming process. This die design concept can provide valuable insights for improving productivity in the wire drawing industry.

The most significant challenge for the practical application of the WD process is the lubrication issue caused by the temperature rise and the resulting surface quality deterioration of the wire and die wear. Although this study, as a fundamental investigation of the WD process, did not consider the effect of die temperature on lubrication, die wear, and the wire’s surface quality, the author plans to conduct more detailed research on these aspects in future studies for practical application. In addition, further research is needed to ensure that the expansion of the die and wire caused by increased die temperature does not affect the accuracy of the wire diameter.

## 5. Conclusions

Based on a comparative study of the influence of the die temperatures, such as CD and WD, on the wire drawing behavior, the following conclusions were derived:

1. Drawability as well as the homogeneity of the microstructure and mechanical properties in TWIP steel were improved using a temperature gradient along the radial direction of the wire using a WD of 400 °C. A higher temperature of about 300 °C at the surface region of the wire with the WD suppressed the twinning rate at the surface region owing to the increase in the SFE from 34 to 55 mJ/m^2^, leading to a uniform twinning rate along the wire’s radial direction compared with the CD wire, finally, resulting in an improvement of the microstructural homogeneity and drawability of the wire.
2. The steel wire subjected to the WD process exhibited an approximately 33% higher drawability compared to that of the conventional CD process. However, the hardness of the WD wire slightly decreased compared to the CD wire.
3. This die design concept can provide valuable insights for improving productivity in the wire drawing industry. In addition, the general conclusion was derived that controlling the SFE within an area of the workpiece by tailoring the temperature can improve the formability in TWIP steels during the plastic forming process.

## Figures and Tables

**Table 1:** Analyzed chemical composition and calculated SFE of present TWIP steel.

	Chemical Composition (wt.%)			SFE (mJ/m^2^)
C	Mn	Al	Fe	
0.91	19.9	1.02	Balance	29.5

**Table 2:** Detailed process conditions for present wire drawing test.

Number of Pass	Diameter (mm)	Die Angle (°)	RA Per Pass (%)	RA in Total (%)	Nominal Drawing Strain	Initial Die Temperature (°C)	
						CD Process	WD Process
-	13.00	-	-	0	0	-	-
1	11.63	12	19.97	19.97	0.22	26	400
2	10.40	12	20.03	36.00	0.45	26	400
3	9.30	12	20.04	48.82	0.67	26	400
4	8.32	12	19.96	59.04	0.89	26	400
5	7.44	12	20.04	67.25	1.17	26	400
6	6.66	12	19.87	73.75	1.34	26	400
7	5.95	12	20.18	79.05	1.56	26	400
8	5.30	12	20.66	83.38	1.79	26	400
9	4.75	12	19.68	86.65	2.01	26	400

## Data Availability

The original contributions presented in this study are included in the article. Further inquiries can be directed to the corresponding author.

## Nomenclature

*A*	area of wire (mm^2^)
*b*_*twin*_	total length of twin boundary in measured area (mm)
*c*_*p*_	specific heat capacity (J/kg°C)
*D*	diameter of wire (mm)
*k*	thermal conductivity (W/m°C)
*K*	strain hardening coefficient (MPa)
L_*d*_	deformation zone length (mm)
*n*	strain hardening exponent
*R*_*mea*_	total measured area (mm^2^)
R_*twin*_	relative twin density (mm/mm^2^)
*T*	temperature (°C)
*V*	drawing speed (mm/s)
Greek symbols	
*α*	semi-die angle (°)
*β*	fraction factor between mechanical work and heat energy
*ε*	strain
$\dot{\varepsilon }$	strain rate (1/s)
*ε*_N_	nominal drawing strain
*σ*	stress (MPa)
*ρ*	density (kg/m^3^)
Subscript	
i	initial value of wire
f	final value of wire
